# Coupling TOR to the Cell Cycle by the Greatwall–Endosulfine–PP2A-B55 Pathway

**DOI:** 10.3390/biom7030059

**Published:** 2017-08-04

**Authors:** Livia Pérez-Hidalgo, Sergio Moreno

**Affiliations:** 1 Institute of Functional Biology and Genomics (IBFG), CSIC/University of Salamanca, 37007 Salamanca, Spain; 2 Institute of Biomedical Research of Salamanca (IBSAL), University Hospital of Salamanca, 37007 Salamanca, Spain

**Keywords:** cell growth, cell cycle, TOR, Greatwall, Endosulfine, PP2A-B55

## Abstract

Cell growth and division are two processes tightly coupled in proliferating cells. While Target of Rapamycin (TOR) is the master regulator of growth, the cell cycle is dictated by the activity of the cyclin-dependent kinases (CDKs). A long-standing question in cell biology is how these processes may be connected. Recent work has highlighted that regulating the phosphatases that revert CDK phosphorylations is as important as regulating the CDKs for cell cycle progression. At mitosis, maintaining a low level of protein phosphatase 2A (PP2A)-B55 activity is essential for CDK substrates to achieve the correct level of phosphorylation. The conserved Greatwall–Endosulfine pathway has been shown to be required for PP2A-B55 inhibition at mitosis in yeasts and multicellular organisms. Interestingly, in yeasts, the Greatwall–Endosulfine pathway is negatively regulated by TOR Complex 1 (TORC1). Moreover, Greatwall–Endosulfine activation upon TORC1 inhibition has been shown to regulate the progression of the cell cycle at different points: the G1 phase in budding yeast, the G2/M transition and the differentiation response in fission yeast, and the entry into quiescence in both budding and fission yeasts. In this review, we discuss the recent findings on how the Greatwall–Endosulfine pathway may provide a connection between cell growth and the cell cycle machinery.

## 1. Introduction

Cell growth and cell cycle progression occur in a separate but coordinated manner to promote cell proliferation. Mutants of the cell cycle machinery arrest the cell cycle but do not prevent growth, leading to cells with an increased cell size, which indicates that growth and the cell cycle progression are regulated by different pathways [1,2,3]. On the other hand, in fission yeast, cells need to reach a critical size to enter mitosis: a minimal size that can be modified in response to environmental changes [4]. These observations suggest that connections must exist between the pathways that regulate cell growth and the cell cycle machinery.

The highly conserved Target of Rapamycin (TOR) kinase plays a central role in the control of growth and proliferation in eukaryotes. The TOR kinase assembles into two multiprotein complexes, TOR Complex 1 (TORC1) and TOR Complex 2 (TORC2), whose functions are also highly conserved through evolution. In contrast to mammals, for which a single mTOR kinase is the catalytic subunit of two multiprotein complexes, mTORC1 and mTORC2, budding and fission yeast complexes contain one of two kinases, Tor1 or Tor2. In addition, these complexes contain specific subunits, Raptor in TORC1, and Rictor, Sin1 and Protor/Bit61 in TORC2, and share subunits such as Deptor (in mammals) and Lst8/Wat1 [5]. In *Saccharomyces cerevisiae*, Tor1 is the main kinase subunit of the TORC1 complex, but Tor2 can also be found in a minor proportion, while TORC2 only contains Tor2 [6]. In *Schizosaccharomyces pombe*, the nomenclature can be misleading, as the Tor2 kinase in association with Mip1 (Raptor in mammalian cells) forms the TORC1 complex. Tor1, on the other hand, mainly interacts with Ste20 (Rictor in mammalian cells) and forms the TORC2 complex [7,8,9] (Table 1).

The TOR pathway was discovered in yeast as the target of the macrolide antibiotic rapamycin. Rapamycin binds to the prolyl isomerase FKBP12 (FK506-binding protein 1A, 12 kDa), forming a complex that specifically inhibits most of the functions of TORC1. By contrast, TORC2 is insensitive to this drug. The structural basis of this insensitivity has recently been shown to be the blocking of the FKBP12–rapamycin complex binding site by the Rictor subunit in the TORC2 complex [10]. As a central controller of growth, TORC1 is sensitive to nutrients, growth factors and hormones, and promotes anabolic processes such as the synthesis of ribosomes, transcription and translation. At the same time, TORC1 inhibits catabolic processes such as autophagy. Interestingly, recent studies have shown that although TORC1 regulates protein synthesis and autophagy, these processes are able to regulate TORC1. These feedback loops help to maintain cellular homeostasis [11]. The functions of TORC2 range from the regulation of the actin cytoskeleton, endocytosis, lipid biosynthesis and stress responses in budding yeast [12,13,14], to growth under starvation conditions, responses to different kinds of stress and DNA damage, cytokinesis, gene silencing and the maintenance of telomeres in fission yeast [15,16,17,18]. Many of the functions of TORC1 and TORC2 have been uncovered through the analysis of the AGC kinases acting downstream: S6 kinase (S6K) in the case of TORC1 and Akt in the case of TORC2 [19,20,21,22,23,24].

Progression through the cell cycle depends on oscillations of cyclin-dependent kinase (CDK)–cyclin activity. According to the quantitative model of the cell cycle, a reduction of CDK activity to minimal levels is required for the assembly of DNA pre-replication complexes in G1, intermediate levels of CDK activity are required for DNA replication (S phase) and high levels for mitosis [25]. The classical model that explained these oscillations involved the association of the CDK with different cyclins at different stages of the cell cycle, in addition to the regulation of the phosphorylation of the CDK by the inhibitory kinases Wee1 and Myt1 and the activating phosphatase Cdc25. This model has been challenged by recent studies that show that only one CDK–cyclin complex (Cdk1–cyclin B) is sufficient to run the cell cycle [26], and that properties of the substrates determine the order of phosphorylation—good substrates being phosphorylated early (with lower CDK activity) and bad substrates being phosphorylated late (with higher CDK activity) [27]. That which makes a substrate efficient or inefficient seems to be not only the surrounding amino acid sequence, but also the identity of the phosphorylated residue itself. Thus, serines are preferred by CDKs, but they are inefficiently dephosphorylated by the phosphatase PP2A-B55, resulting in early phosphorylation. By contrast, threonines are the preferred sites of PP2A-B55 and are phosphorylated late [28,29].

Numerous recent studies highlight the importance of the phosphatases that reverse the phosphorylations of CDK substrates for mitotic entry, progression, and exit from mitosis [30,31,32,33,34]. The net phosphorylation of CDK substrates depends on the balance between CDK activity and counteracting phosphatases. In order to reach maximal phosphorylation of CDK substrates at mitosis, the main phosphatase that reverses these phosphorylations, PP2A in complex with the regulatory subunit B55, must be inhibited. The kinase responsible for this inhibition, Greatwall, was first described in *Drosophila*, and its absence results in defects in chromosome condensation and mitotic progression [35]. Experiments in *Xenopus*, for which Greatwall is required for mitotic entry [36], show that the inhibition of PP2A-B55 by Greatwall is not direct, but occurs through small proteins called Endosulfines (ENSA and ARPP-19), which are phosphorylated by Greatwall to become potent and specific inhibitors of the PP2A-B55 complexes [37,38]. Further work showed that the Greatwall–Endosulfine pathway is conserved in mammalian cells, where inactivation of Greatwall (MASTL in human cells) results in defects in mitosis and cytokinesis [39,40,41]. Interestingly, the budding yeast orthologue of Greatwall Rim15 was discovered prior to Greatwall in *Drosophila* as a protein negatively regulated by TORC1 and protein kinase A (PKA) that is required to stimulate meiotic gene expression and also for entry into the G0 phase [42,43,44]. In 2010, de Virgilio and colleagues showed that Igo1 and Igo2 are the orthologues of Endosulfines in budding yeast and, similarly to Rim15, are required for entry into quiescence [45]. In fission yeast, the pathway is conserved and, as in budding yeast, it seems to be negatively regulated by TORC1 [46]. 

In this review, we will discuss the recent findings on the connections between TORC1 and TORC2 and the cell cycle, with particular focus on the functions channelled through the TORC1–Greatwall–Endosulfine–PP2A-B55 pathway in budding and fission yeast.

## 2. Regulation of the G2/M Transition and Cell Size in Fission Yeast

*S. pombe* is an excellent model system for the analysis of the cell cycle and its connection with cell growth. Fission yeast cells growing in a nitrogen-rich medium have a long G2 phase and a very short G1 phase. Growth occurs during G2, and there is only an active size control at the G2/M transition, while the G1/S control is cryptic. When cells are transferred to a nitrogen-poor medium, they enter mitosis earlier at a reduced cell size, and the G1/S control is activated and the G1 phase extends because the cells do not have the minimal size and need to grow before entering into a new cell cycle. As fission yeast cells are rod shaped and grow by tip elongation, the cell length at division is an indicator of the duration of the G2 phase [4]. In fission yeast, inactivation of TORC1 with a thermosensitive *tor2* mutant mimics nitrogen starvation, inducing mitotic entry at a smaller cell size [7,8,47]. This smaller cell size has also been observed in tissue cultures cells after TORC1 inactivation and after inactivation of the TORC1 target S6K [3,48]. Moreover, in *Drosophila* and mice, the inactivation of the S6K leads to smaller organisms [49,50]. Interestingly, the overexpression of S6K in mammalian cells and of the S6K orthologue in fission yeast, *sck2^+^*, induces cell growth, likely through the activation of biosynthetic pathways [3,51,52,53]. The following questions emerge from this observation: is TORC1 only regulating growth, and is the reduction of cell size after TORC1 inactivation just a consequence of the inactivation of anabolic processes and the activation of autophagy? Or is TORC1 also regulating the cell cycle progression by interacting with the cell cycle machinery, modifying the cell size threshold for cell division? Alternatively, the transcriptional and translational regulation of specific components of the cell cycle machinery, such as the Cdc25 phosphatase in fission yeast, could be the final target that connects TORC1 with cell cycle progression [54,55].

**Figure 1:**
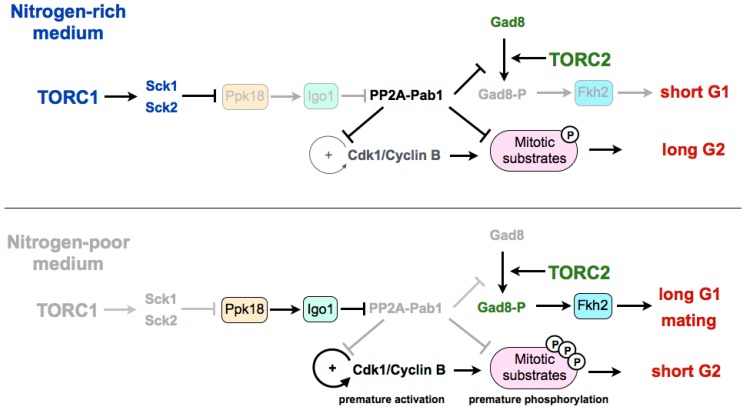
Current model of the nutritional regulation of the cell cycle by the TORC1–Greatwall–Endosulfine–PP2A-B55 pathway. In nitrogen-poor media, PP2A-Pab1 is inactivated by the Ppk18–Igo1 pathway. Inactivation of PP2A-Pab1: (1) prevents the dephosphorylation of Cdk1–cyclin B target proteins, including Cdc25 and Wee1 (autocatalytic loop) leading to premature entry into mitosis; (2) prevents the dephosphorylation and inactivation of Gad8, leading to the extension of the G1 phase and sexual differentiation. TORC1: Tor complex 1. Sck1 and Sck2: S6 kinases. Ppk18: Greatwall. Igo1: Endosulfine. PP2A-Pab1: PP2A-B55. Gad8: Akt. Fkh2: Transcription factor forkhead 2.

Experiments in fission yeast suggest that TORC1 may be modulating the G2/M transition and the cell size at division by regulating the Greatwall–Endosulfine pathway, which inhibits the CDK-counteracting phosphatase PP2A-B55 (Figure 1). In a rich medium, TORC1 promotes cell size increase during G2 through inhibition of the Greatwall–Endosulfine pathway. This inhibition results in the activation of the PP2A-B55 phosphatase, which counteracts the activity of the Cdk1–cyclin B complex and delays mitosis. On the contrary, in media with low nutrients—both minimal media without nitrogen or a medium with a poor nitrogen source, such as phenylalanine—TORC1 is inactivated, leading to the activation of the Greatwall–Endosulfine pathway and PP2A-B55 inhibition. Reduction of the phosphatase activity allows the cells to reach the level of CDK substrate phosphorylation required to enter mitosis prematurely, and thus cells divide at a reduced cell size [46]. According to this, cells lacking Igo1 (fission yeast Endosulfine) or Ppk18 (fission yeast Greatwall) are unable to accelerate mitotic entry and do not reduce their size in a nitrogen-poor medium. By contrast, moderate overexpression of *ppk18^+^* promotes mitotic entry in a rich medium, mimicking the phenotype of the deletion of the catalytic subunit of PP2A (*ppa2^+^*) or the downregulation of the B55 regulatory subunit (*pab1^+^*) [56,57,58]. In addition, Igo1 is required for the reduction of cell size after inactivation of TORC1 [46]. 

## 3. Coordination of TORC1 and TORC2 in the Differentiation Response

In fission yeast, the differentiation response requires the reduction of nutrients in the medium as well as the presence of cells of both mating types [59]. The limitation of nutrients leads to the inactivation of TORC1, and this inactivation has been shown to increase mating, not only through the extension of the G1 phase, but also because TORC1 inactivation induces the set of genes required for the mating response [7,47]. On the other hand, TORC2 is essential under starvation and stress conditions. Cells deleted for *tor1^+^*, as for cells deleted for its target *gad8^+^* (orthologous of Akt in mammalian cells), are unable to arrest in G1, which results in sterility [60,61]. The transcription factor Fkh2, required for the expression of mating genes, has been shown to be a substrate of the TORC2 effector kinase Gad8 in vitro [15,62,63]. Recently it has been shown that in fission yeast, the differentiation response is mediated by active Gad8 phosphorylated on serine 546. This phosphorylation event is also counteracted by PP2A-B55. Therefore, upon TORC1 inhibition, the Ppk18–Igo1 signalling module is required to reduce PP2A-B55 to allow the accumulation of active phospho-Gad8–S546 [64] (Figure 1). According to this, the transcriptional profile of a thermosensitive *tor2* mutant significantly overlaps with the profile of cells lacking Pab1 (B55 regulatory subunit of PP2A). Consistent with a positive role of Ppk18 and Igo1 on the differentiation response, cells deleted for either of these genes show an inability to arrest in G1 in the absence of nitrogen and a reduction in sporulation [46]. By contrast, PP2A-B55 plays a negative role in sporulation, and the deletion of *pab1^+^*, encoding the B55 subunit, is hyperfertile, and rescues the sporulation defect of cells overexpressing *tor2^+^* or cells deleted for the TORC1 inhibitor *tsc2^+^*. Martin et al. show that the hyperfertility of the *pab1* mutant depends on the phosphorylation of Gad8, as a S546-phosphonull mutant of Gad8 in *pab1^+^*-deleted cells decreases the expression of *mei2* and reduces its conjugation efficiency [64]. Moreover, the PP2A-B55 complex is able to reduce the phosphorylation of this residue in vitro. The connection between TORC1 and TORC2 through both the Ppk18–Igo1–PP2A-B55 pathway and the phosphorylation of Gad8 may explain the long-standing observation that TORC1 and TORC2 complexes play opposite roles with regard to sexual differentiation, with TORC1 repressing, in a rich medium, the expression of genes required for sporulation (mutations of *tor2^+^* are hyperfertile), and TORC2 being required for the mating response in a poor medium (cells deleted for *tor1^+^* or *gad8^+^* are sterile) [65]. Interestingly, in mammalian cells, for which no connection has yet been established between the Greatwall–Endosulfine pathway and TORC1, it has been recently shown that overexpression of Greatwall increases the phosphorylation and activation of Akt (the mammalian orthologue of Gad8) through the degradation of a phosphatase [66]. Despite the fact that this function of human Greatwall is independent of ENSA and involves a phosphatase different from PP2A, both models share intriguing similarities.

In budding yeast, the differentiation response is negatively regulated by TORC1 and PKA, and also requires the activation of the Rim15–Igo1/2–PP2A-Cdc55 module [67]. Cells lacking both *IGO1* and *IGO2*, as for cells deleted for *RIM15*, undergo inefficient premeiotic S phase and gametogenesis. Phosphorylation of Igo1–S64 occurs at meiotic entry and subsequently disappears, suggesting that its function is restricted to early stages [67].

## 4. Regulation of G1/S in Budding Yeast

### 4.1. Stabilization of Sic1

**Figure 2:**
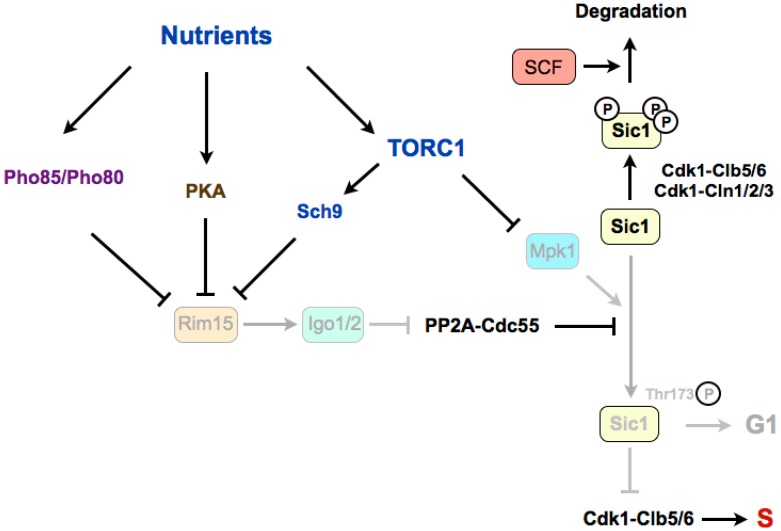
The Greatwall–Endosulfine–PP2A-B55 pathway connects TORC1 with the stability of the Cdk1 inhibitor Sic1 in budding yeast. Sic1 stabilization in poor nutrients requires the phosphorylation of Sic1 promoted by the Mpk1 kinase and by the Rim15–Igo1/2-dependent inactivation of PP2A-Cdc55. S: S-phase. SCF: Skp1–Cul1–F-box protein.

In budding yeast, rapamycin treatment leads to G1 arrest through a double mechanism involving the downregulation of G1 cyclins and the accumulation of the Cdk1–cyclin inhibitor Sic1, which inhibits the Cdk1–Clb5,6 complexes that are required for the onset of DNA replication [68,69]. TORC1 stimulates transcription and translation of G1 cyclins, thus activating Cdk1–cyclin complexes required for the G1/S transition. In this sense, it has been shown that in the presence of rapamycin, the inhibition of the translation of cyclin Cln3 is linked to the presence of upstream open reading frames (uORFs) in the 5’ untranslated region (UTR) of its mRNA [70,71]. The stability of Sic1 is regulated by phosphorylation (Figure 2). When TORC1 is active, Cdk1–cyclins phosphorylate Sic1, and phosphorylated Sic1 binds to SCF (Skp1–Cul1–F-box)–Cdc4 E3 ubiquitin-ligase, which catalyzes Sic1 ubiquitination and targets it for degradation by the proteasome, thus leading to a reduction of Sic1 levels [72,73,74]. When nutrients are limited, or in the presence of rapamycin, TORC1 is inactivated and G1 cyclin levels decrease, which relieves the negative regulation of Cdk1–cyclin complexes on Sic1. Stabilization of Sic1 in a poor medium, in addition to the downregulation of G1 cyclins, requires the phosphorylation of Sic1 on a specific residue, Thr173 [69]. Recently, it has been shown that this residue is phosphorylated by the MAP kinase Mpk1 in rapamycin, and is dephosphorylated by PP2A-B55 in a rich medium [75]. TORC1 inactivation promotes the stabilizing C-terminal phosphorylation of Sic1 through the activation of the Mpk1 kinase and the activation of the Greatwall–Endosulfine pathway, which inhibits the PP2A-B55 complexes. According to this, mutation of Thr173 to alanine destabilizes Sic1 and compromises the G1 arrest in rapamycin, as for deletions of *RIM15* (Greatwall) or *IGO1* and *IGO2* (Endosulfines). Therefore, Rim15–Igo1/2 preserves phosphorylation of Sic1 on Thr173 to prevent its degradation [75]. The molecular mechanism of this stabilization has recently been uncovered. The key residue at the C-terminus of Sic1, Thr173, once phosphorylated by Mpk1, behaves as a docking site for Cks1, and this association prevents the inhibitory phosphorylation of Sic1 by Cdk1–Clb5 and the subsequent ubiquitin flagging and proteasome degradation [76]. Moreover, this conformation also promotes the inhibition of Cdk1–Clb5 complexes by Sic1 through the interaction of Clb5 with the RXL motif at the C-terminus of Sic1, converting Sic1 from a target to an inhibitor of Cdk1–Clb5 complexes. Interestingly, the phosphorylation of this residue also occurs at low levels in proliferating cells and it is cell-cycle regulated, increasing in G1. This oscillation has uncovered that, despite negative regulation by TORC1–Sch9, Rim15 is active in a rich medium, and fluctuates during the cell cycle because of negative regulation by Cdk1–Cln1,2,3. At G1, Rim15 activity is higher and PP2A-B55 is inactivated, thus leading to Sic1–Thr173 phosphorylation and protein stabilization. At the G1/S transition, Cdk1–Cln1,2,3 phosphorylate and inhibit Rim15, possibly in parallel to phosphorylation by the CDK kinase Pho85, promoting its cytoplasmic retention by 14-3-3 proteins. Rim15 inhibition permits activation of PP2A-B55, which dephosphorylates Sic1–Thr173, leading to its destabilization [76]. Although this phosphorylation site is not conserved in Rum1 (the fission yeast orthologue of Sic1), a similar mechanism may occur in fission yeast, as Rum1 protein is also stabilised in G1.

### 4.2. Inactivation of Whi5

**Figure 3:**
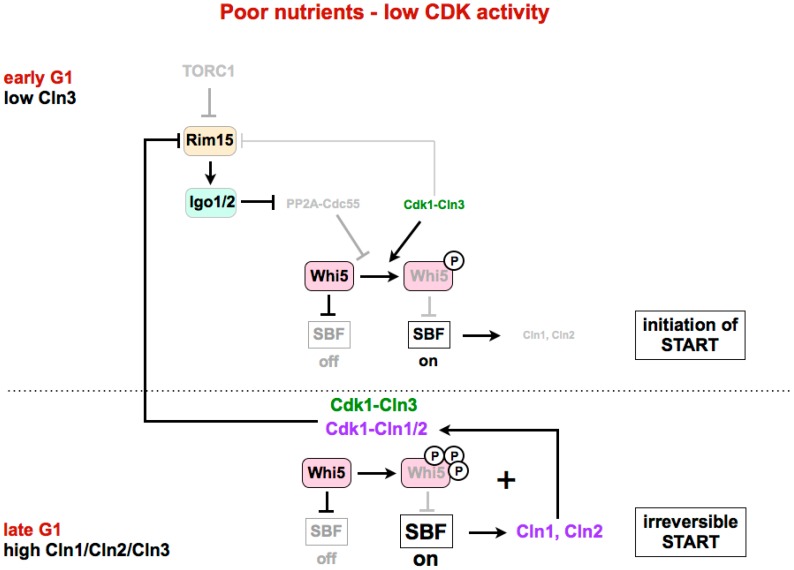
The Greatwall–Endosulfine–PP2A-B55 pathway connects TORC1 with the activity of the SBF repressor Whi5. In poor nutrients or in a medium with low CDK ctivity, activation of the Rim15–Igo1/2 pathway and subsequent inhibition of PP2A-B55 is required for the phosphorylation of Whi5 by Cdk1–Cln3 to initiate activation of SBF. Later in G1, expression of Cln1 and Cln2 by SBF, together with Cln3, leads to complete phosphorylation and inhibition of Whi5, and permits full activation of SBF. START: cell cycle transition in G1 where cells commit themselves to one round of cell division. SBF: SCB (Swi4/6 cell cycle box)-binding factor.

Recently, it has been shown that the Greatwall–Endosulfine pathway could also be regulating START in budding yeast [77]. Similarly to other eukaryotes, in *S. cerevisiae*, Cdk1 activity is required for the G1/S transition. In budding yeast, Cdk1 associated with the G1 cyclin Cln3 determines the cell size at START and helps to trigger the expression of late G1 cyclins Cln1 and Cln2. In late G1, all three cyclins, Cln1, Cln2 and Cln3, collaborate to make START transition irreversible. However, the current model does not explain why, under nutrient limitation and despite the lower levels of Cdk1–Cln3, budding yeast cells pass START at a smaller cell size. The key CDK substrate that needs to be phosphorylated for G1/S transition is Whi5 (functionally related to mammalian Rb), which, in its dephosphorylated form, is a repressor of SBF (E2F in mammalian cells), a transcription factor required for START. The inhibitory phosphorylation of Whi5 is carried out by Cdk1–Cln1,2,3, and is reversed by PP2A-B55. Talarek et al. have recently shown that in early G1, when Cdk1–Cln3 activity is low, Rim15 is active in proliferating cells, and phosphorylates and activates Igo1/2, thus leading to the inhibition of PP2A-B55 (Figure 3, upper part) [77]. This would permit the initial accumulation of the phosphorylated and inactive form of Whi5, and the initiation of SBF transcription. The expression of Cln1 and Cln2 by SBF, together with Cln3, leads to an increase of Cdk1 activity and Whi5 phosphorylation, making the START transition irreversible (Figure 3, lower part).

The activity of Rim15 in proliferating cells is high in early G1, when Cdk1–Cln3 activity is low, but decreases during G1 because Cdk1–Cln1,2,3 activity, as shown by Moreno-Torres et al. [76], inhibits Rim15. However, under normal laboratory conditions, that is, in a rich medium, the CDK activity is high, and there is no need to inhibit PP2A for the complete phosphorylation of CDK substrates. According to this, in a rich medium, the cell size at the START transition in cells lacking *IGO1/IGO2* is very similar to that of the wild type. However, when the CDK activity is lower (in cells expressing a hypomorphic allele of Cdk1) or when cells are grown in a poor medium (glycerol/lactate instead of glucose), the Rim15–Igo1/2 pathway is required to facilitate Whi5 phosphorylation and promote START at a smaller cell size. As a result, in these conditions of a poor medium or low Cdk1 activity, the deletion of *IGO1/IGO2* shows an increased cell size at START.

Altogether, and according to the models proposed by Moreno-Torres et al. and Talarek et al., the Greatwall–Endosulfine pathway needs to be finely tuned in early G1 cells and in cells growing in a poor medium, to promote the stabilization of the CDK inhibitor Sic1, and to initiate Whi5 phosphorylation and help promote subsequent START [75,76,77].

## 5. TORC1–Greatwall–Endosulfine in Quiescence in Budding and Fission Yeast

In eukaryotes, entry into quiescence (G0 phase) occurs in the absence of growth factors, hormones or nutrients, which stimulate cell proliferation. In yeast, the quiescence programme is induced by a limitation of nutrients, and involves a general down-regulation of protein synthesis, and transcription and the activation of several metabolic pathways and stress responses that are essential for the maintenance of survival during the stationary phase [78,79]. In budding yeast, a drop of the carbon and nitrogen sources results in PKA and TORC1 inactivation, respectively. PKA and TORC1, in addition to Pho85–Pho80, which is sensitive to phosphate levels, phosphorylate and inhibit Rim15 under nutrient-rich conditions by promoting the exclusion of Rim15 from the nucleus through the interaction with 14-3-3 proteins (in the case of Sch9 and Pho85 phosphorylation) or by directly inhibiting its kinase activity (in the case of PKA phosphorylation) [43,44,80,81]. Rim15 activation under starvation conditions, and the subsequent phosphorylation of Igo1/Igo2 and inhibition of PP2A-B55, results in the activation of the quiescence program through the activation of transcription by specific transcription factors (Msn2/4, Gis1 and Hsf1), and the stabilization of specific mRNAs. For the induction of transcription, inactivation of PP2A-B55 promotes the accumulation of the Gis1 transcription factor in its phosphorylated state and its recruitment to promoters of specific genes [82]. In addition, Rim15 may be regulating Msn2/4 and Hsf1 transcription factors by direct phosphorylation [83]. For mRNA stabilization, phosphorylated Igo1/Igo2 protect specific nutrient-regulated mRNAs required for the stationary phase from degradation by interfering with the 5’-to-3’ mRNA decay pathway during initiation of the G0 program [45,84]. Moreover, the suppression of the mRNA stabilization defect in the *rim15*∆ and *igo1*∆ *igo2*∆ mutants by the deletion of *CDC55* (B55) suggests that stabilization requires inactivation of PP2A-B55 [85]. Altogether, TORC1 inactivation leads to the activation of Rim15 and Igo1/Igo2 phosphorylation, and phosphorylated Igo1/2 inhibit PP2A-B55, leading to transcription and stabilization of specific nutrient-regulated mRNAs. Consequently, *rim15*∆ and *igo1*∆ *igo2*∆ mutants show a dramatic reduction of chronological lifespan [45]. 

Interestingly, the orthologues of Greatwall in fission yeast, Ppk18 and Cek1, have been shown to be also involved in lifespan regulation [86].

## 6. Conclusions and Future Perspectives

In this review, we have discussed different aspects of TORC1 and TORC2 that affect the cell cycle. Recent research in many laboratories has focused on the phosphatase PP2A-B55 and on the Greatwall–Endosulfine pathway, whose inhibitory activity over PP2A-B55 contributes to the regulation of cell cycle progression. In budding and fission yeast, the Greatwall–Endosulfine pathway is negatively regulated by TORC1, linking nutrient signalling with the cell cycle. The use of different model systems with different cell cycles has favoured the uncovering of different aspects of this regulation. Budding yeast, for which the main point of regulation is at G1/S, has revealed that this pathway is important for the stabilization of the CDK inhibitor Sic1 and for the initial phosphorylation of Whi5 in a medium with low nutrients. The fission yeast, with the main control point at G2/M, has uncovered an important role in regulating G2/M and the cell size in media with poor nutrients. The fission yeast model establishes a link between mammalian cells, for which the Greatwall–Endosulfine pathway controls mitosis, and budding yeast, for which the pathway responds to nutrients but whose main control is at G1/S. In addition, in fission yeast, the Greatwall–Endosulfine pathway has given a molecular explanation to the question of how TORC1 and TORC2 are connected and play opposing roles in the differentiation response. 

Many questions still remain, especially regarding the regulation of TORC2. First, it is unknown how TORC2 regulates the extension of the G1 phase of the cell cycle. Interestingly, it has been observed that the expression of the CDK inhibitor Rum1 is reduced in *tor1* and *gad8* mutants [15]. The consequent increase in CDK activity could be contributing to the defective G1 arrest, as well as to the increased genome instability exhibited by TORC2 mutants [15,16,17]. Furthermore, it has been recently shown that the kinase downstream TORC2, Gad8, is required for efficient binding of the MluI cell cycle box binding factor (MBF) complex to promoters, and for the expression of MBF target genes under replication stress [87]. This defective transcriptional activation may contribute to the increased sensitivity to chronic replication stress of TORC2 mutants [17]. Interestingly, the function of TORC2 in the maintenance of genome stability is conserved in budding yeast, and extends to mammalian cells [14,88,89].

Besides Greatwall–Endosulfine, other pathways may be involved in the crosstalk between cell growth and the cell cycle machinery. For example, in budding yeast, TORC1 has been shown to promote cell cycle progression at G2/M through the destabilization of the CDK inhibitor Swe1 [90]. In mammalian cells, a CDK-dependent control of the TORC1 inhibitor TSC1–TSC2 has been suggested [91,92,93]. It would be interesting to explore whether these pathways are conserved in other organisms. Future work will continue to identify common pathways behind singularities.

## Figures and Tables

**Table 1:** Composition of the TOR Complex 1 (TORC1) and TOR Complex 2 (TORC2) in budding yeast, fission yeast and mammalian cells.

	*S. cerevisiae*	*S. pombe*	Mammals
TORC1	Tor1 or Tor2	Tor2	mTOR
	Kog1 (Raptor)	Mip1 (Raptor)	Raptor
	—	—	Deptor
	Lst8	Wat1	mLst8
TORC2	Tor2	Tor1	mTOR
	Avo3 (Rictor)	Ste20 (Rictor)	Rictor
	Avo1	Sin1	mSin1
	Bit61	Bit61	Protor
	—	—	Deptor
	Lst8	Wat1	mLst8
